# Estimation of Cancer Burden Attributable to Infection in Asia

**DOI:** 10.2188/jea.JE20140215

**Published:** 2015-11-05

**Authors:** He Huang, Xiao-Feng Hu, Fang-Hui Zhao, Suzanne M. Garland, Neerja Bhatla, You-Lin Qiao

**Affiliations:** 1 Department of Cancer Epidemiology, Cancer Institute, Chinese Academy of Medical Sciences & Peking Union Medical College, Beijing, China; 2 Department of Prevention and Control of Communicable Disease, Guizhou Center for Disease Control and Prevention, Guiyang, Guizhou, China; 3 Shijingshan District Center for Disease Control and Prevention, Beijing, China; 4 The Microbiology and Infectious Diseases Department, Royal Women’s Hospital, and the Department of Obstetrics and Gynecology, University of Melbourne, Melbourne, Australia; 5 Department of Obstetrics & Gynaecology, All India Institute of Medical Sciences, Ansari Nagar, New Delhi, India

**Keywords:** cancer burden, infection, population attributable fraction, Asia

## Abstract

**Background:**

Some infectious agents have been shown to be human carcinogens. The current study focused on estimation of cancer burden attributable to infection in different regions of Asia.

**Methods:**

By systematically reviewing previous studies of the infection prevalence data of 13 countries in Asia and relative risks of specific cancers, we calculated the population attributable fraction of carcinogenic infections. Using data from GLOBOCAN 2012, the overall country-specific and gender-specific number of new cancer cases and deaths resulting from infection were estimated.

**Results:**

Across 13 principal Asian countries, the average prevalence and range was 6.6% (0.5% in Japanese women to 15.0% in Vietnamese men) for hepatitis B virus (HBV), 2.6% (0.3% in Iran to 5.1% in Saudi Arabia) for hepatitis C virus (HCV), 7.9% (2.8% in Pakistan to 17.7% in China) for human papillomavirus (HPV), and 61.8% (12.8% in Indonesia to 91.7% in Bangladesh) for *Helicobacter pylori* (HP). The estimated total number of cancer cases and deaths caused by infection in these 13 countries were 1 212 026 (19.6% of all new cancer cases) and 908 549 (22.0% of all deaths from cancer). The fractions of cancer incidence attributable to infection were 19.7% and 19.5% in men and women, respectively. The percentages of cancer deaths attributable to infection were 21.9% and 22.1% in men and women, respectively. Among the main infectious agents, HP was responsible for 31.5% of infection-related cancer cases and 32.8% of infection-related cancer deaths, followed by HBV (28.6% of new cases and 23.8% of deaths), HPV (22.0% of new cases and 27.3% of deaths), and HCV (12.2% of new cases and 10.6% of deaths).

**Conclusions:**

Approximately one quarter of all cancer cases and deaths were infection-associated in Asia, which could be effectively prevented if appropriate long-term controls of infectious agents were applied.

## INTRODUCTION

Chronic infection with potentially carcinogenic agents is recognized as a major risk factor of human cancer^1^^,^^2^ and was estimated to be responsible for around 2 million new cancer cases worldwide in 2008. The overall population attributable fraction (PAF) for infectious agents was 16.1% worldwide,^3^ indicating that 16.1% of new cancer cases could be prevented by elimination of such infectious agents. This fraction is higher in developing countries (22.9%) than in developed countries (7.4%).

Asia, which contains over 60% of the world’s population, plays an increasingly important role in international economics and trade. As a part of global analysis, the burden of infection-associated cancer in Asia has been estimated.^3^^,^^4^ While the diversity of environment, culture, and economics in different countries contributes to the variety of infectious agents and their prevalence in the general population, the effects of those factors have not been revealed in previous studies.

The aim of the current study is to assess the cancer burden attributable to infection in 13 Asian countries based on review of nation-specific prevalence of carcinogenic infectious agents and comparison of risk estimates between exposure and infection-associated cancer.

## METHODS

### Geographical areas

As residential environment, social-economic status, habits, and culture vary in different places among different groups of people, we chose countries and regions with the largest population sizes to obtain reliable and stable estimates, while also taking population representativeness into consideration. With the population data of almost all Asian countries and regions available in GLOBOCAN 2012, we chose a cut-off point of 30 million. As a consequence, 13 countries were included and divided into four areas: East Asia (Korea, China, and Japan), Southeast Asia (Indonesia, the Philippines, Thailand, and Vietnam), Middle South Asia (Bangladesh, India, Iran, and Pakistan), and West Asia (Turkey and Saudi-Arabia), following the geographical definition of the 2012 revision of the United Nations’ World Population Prospects.^5^ Although Iraq was on the list of 13 countries, few studies of infection prevalence in Iraq were available. Subsequently, we substituted Saudi Arabia, a smaller West Asian country with a population of 27 million, for Iraq because more information on infection prevalence was available for Saudi Arabia.

### Definition of exposure

Infectious agents with sufficient evidence for their carcinogenicity were defined in an IARC monograph series.^2^ The infectious agents and related cancers used in the current study are listed in Table 1. Other carcinogenetic infectious agents, such as *Schistosoma haematobium* and human T-cell lymphotropic virus type 1 (HTLV-1), were not included in the current analysis because the prevalence of the infectious agents in Asia or the data of cancer incidence and/or mortality were not available in published studies or were only available in low quality studies (eg, studies with small sample sizes or poor study designs).

**Table 1.** List of group 1 carcinogenic biological agents and related cancers

Infection-associated cancers	Group 1 agents
Oral cavity	HPV
Oropharynx	HPV
Nasopharynx	EBV
Noncardia gastric cancer	*Helicobacter pylori*
cardia gastric cancer	*Helicobacter pylori*
Anus	HPV
Liver	
Hepatocellular carcinoma	HBV, HCV
Cholangiocarcinoma	*Clonorchis sinensis*, HBV, HCV, *Opisthorchis viverrini*^a^
Vulva	HPV
Vagina	HPV
Cervix uteri	HPV
Penis	HPV
Hodgkin’s lymphoma	EBV
Non-Hodgkin’s lymphoma	HIV-1, HCV
Burkitt’s lymphoma	EBV
Kaposi’s sarcoma^b^	KSHV/HIV-1

^a^*Opisthorchis viverrini* is only prevalent in Thailand.

^b^The incidence of Kaposi’s sarcoma is low in Asians.

EBV, Epstein-Barr virus; HBV, hepatitis B virus; HCV, hepatitis C virus; HIV-1, human immunodeficiency virus type 1; HPV, human papillomavirus; KSHV, Kaposi’s sarcoma herpes virus (also known as Human herpes virus type 8).

### Prevalence of infectious agents and relative risk of cancers and infectious agents

Searches were conducted in Pubmed, Google Scholar, and Chinese National Knowledge Infrastructure (CNKI) for all published studies in Chinese and English. The keywords of prevalence-oriented searching were “prevalence”, the names of infectious agents, and the country names. Prevalence data published between 1997 and 1999 were collected, fixing the exposure time of infectious factors around 1997. The intervals from exposure to the infectious agents to diagnosis of related cancer were all assumed to be 15 years in the current study.^6^ If the data from 1997–1999 for some countries were not available, we expanded our search to data published between 1995 and 2000. We included case-control studies with large sample sizes only if population-based cross-sectional studies were not available. As a consequence, prevalence data is based on the general population without adjustment for age. In the search for relative risks (RRs), we included keywords of “meta-analysis”, “cohort study”, “case-control study”, and the names of infectious agents and relevant cancers. The priority for data selection was (1) meta-analysis or pooled analysis in Asia; (2) large-scale case-control study in an Asian country; and (3) multinational meta-analysis or meta-analysis from non-Asian countries. For instance, we included (2) only if (1) was not available. The biomarkers of infectious agents were human papillomavirus (HPV) DNA, HPV L1 antibody, serum-antibody against *Helicobacter pylori* (HP), anti-HIV antibody, anti-hepatitis C virus (HCV) antibody, and hepatitis B surface antigen (HBsAg), which are considered to be relatively sensitive and accurate measurements of prevalence in the general population. RRs in these studies are calculated by comparing the probability of developing cancer in an exposed population to that in a non-exposed population (cohort study) or estimated with odds ratios (ORs) in case-control studies. We prioritized results from the latest published studies over older studies because the time effect and newly discovered confounding biases are more likely to be taken into consideration in more recent studies. We assumed that RRs were constant in different countries and adopted generally accepted RRs, which were abstracted from international studies or meta-analysis of worldwide prevalences. Similarly, if the sex-stratified prevalence was not accessible, it was assumed that both sexes were exposed equally to infectious factors, and sex-stratified prevalence was replaced by overall prevalence. The prevalence among patients was adopted when the general prevalence was not available. To account for the differences between general population and patient prevalences, Formula B (which we discuss in the Methods section), was used in PAF calculation.

As 85%–95% of the general population had positive serology tests (IgG) for Epstein-Barr virus (EBV) infection when young,^7^^,^^8^ it was not necessary to match the prevalence of EBV in calculation of cancer burden. As a result, the PAFs of EBV-related cancers, which were calculated from case-series prevalence, were applied.^3^

### Incidence and mortality data of infection-related cancer

The cancer-specific sex-stratified incidence and mortality in each country were obtained from GLOBOCAN 2012 and a cancer register project (cancer incidence of Japan).^9^ The numbers of new cases of cancer and cancer-related deaths that were attributable to infection were estimated by multiplying the overall numbers of new cases of cancer and cancer-related deaths with the corresponding PAF. The data of anal cancer, penile cancer, vulvar cancer, and vaginal cancer were included in the category of others and unspecified cancers in the GLOBOCAN project. Therefore, we referred to *Cancer Incidence in Five Continents Vol. X* (CI5X),^10^ which recorded cancer-sorted incidence data worldwide from 2003 to 2007, to estimate the proportions of these cancers. For countries which were not listed in the CI5X (Bangladesh, Indonesia, Vietnam, Iran, and Saudi Arabia), we assumed geographic similarity of cancer incidence patterns in the same area of Asia. For example, Bangladesh is located in Middle South Asia, so the cancer incidence data was assumed to be consistent with that of Pakistan.

Specific data on incidence and mortality related to noncardia and cardia gastric cancer, hepatocellular carcinoma, and cholangiocarcinoma in Korea was obtained in an original article^11^ because the national registry was of high quality with broader categories than those of GLOBOCAN 2012.

It was assumed that Burkitt’s lymphoma accounted for 2.6% of the total non-Hodgkin’s lymphoma (NHL) cases in East/Southeast Asia (however, the proportion in Japan is assumed to be <0.1%) and 14.8% in West Asia. In developing areas of Asia, noncardia gastric cancer constituted 80.0% and 87.0% of gastric cancers in men and women, respectively, while in developed areas, the respective proportions were 80.0% and 88.0%.^4^ We assumed the proportions of hepatocellular carcinoma and cholangiocarcinoma in liver cancer to be 80% and 15%, respectively. The assumed incidence of cholangiocarcinoma was higher than the world average due to a markedly high prevalence of liver flukes in the study areas.^12^

By applying these findings to Levin’s formula $\text{AF}=\frac{\text{P}\times (\text{RR}-1)}{[\text{P}\times (\text{RR}-1)]+1}$ (Formula A) or $\text{AF}=\frac{\left(\text{RR}-1\right)}{\text{RR}}\text{Pc}$ (Formula B)^13^ (where P is the prevalence of infectious agent in general population, Pc is the prevalence among patients, and RR is the RR of cancer among infectious agents exposed population), PAFs were calculated. Combining the data of cancer incidence and mortality in each country from GLOBOCAN 2012, the overall and specific numbers of new cancer cases and cancer-related mortality caused by infection were estimated. We hypothesized that infection had no effect on survival.

## RESULTS

Table 2 shows the data source of RRs applied in the current study, the biomarkers of each infectious agent, the study design, and the target population of each study. The RRs of human papillomavirus (HPV)-related anal cancer, vulvar cancer, vaginal cancer, and penile cancer were replaced with corresponding generally accepted PAFs because no data for RRs were available.^14^ Oncogenic HPV is the etiological agent of cervical cancer, while EBV and Kaposi’s sarcoma-associated herpes virus/human immunodeficiency virus (KSHV/HIV-1) play vital roles in nasopharynx cancer (100.0% of nasopharynx cancers were assumed to be EBV-related in medium- and high-risk areas, and 90.0% elsewhere^4^) and Kaposi’s sarcoma (the RRs were 97.5 in men and 202.7 in women, respectively).^15^ Therefore, the PAFs of these cancers were assumed to be 100. Information about the prevalence of etiologic agents in the general population of the study countries and the RRs of corresponding cancers, as well as gender-specific prevalence of infection by carcinogenic infectious agents, are shown in Table 3. Table 4 specifies PAFs of infectious agents and cancer sites in different countries among men and women. Table 5 shows total estimated new cases and deaths caused by infection.

**Table 2.** Summary of applied relative risks and data sources

Cancer site	Infectious agent	Relative risk		Data source		
	
		Male	Female	Biomarker	Study design	Study population [reference number]
Oral cavity	HPV	2.0 (1.2–3.4)	2.0 (1.2–3.4)	HPV L1 antibody or HPV-DNA (tumor tissue)	Meta-analysis	International [16] 1656 cases
Oropharynx	HPV	12.3 (5.4–26.4)	12.3 (5.4–26.4)	HPV-DNA (tumor tissue)	Case-control study	USA [17] 100/200
Nasopharynx	EBV	PAF 90^a^	PAF 90^a^	EBV-DNA (tumor tissue)	Review	International [4]
Noncardia stomach	HP	5.9 (3.4–10.3)	5.9 (3.4–10.3)	Anti-HP antibody (blood)	Meta-analysis	International [18]
Cardia stomach	HP	1.6 (1.0–2.5)	1.6 (1.0–2.5)	Anti-HP antibody (blood)	Meta-analysis	International [19]
Anus	HPV	PAF88	PAF88	HPV DNA (tumor tissue)	Meta-analysis	International [20]
Hepatocellular carcinoma	HBV	18.1 (10.7–28.8)	18.1 (10.7–28.8)	HBsAg (blood)	Meta-analysis	China [21]
	HCV	13.1 (5.3–27.0)	13.1 (5.3–27.0)	Anti-HCV antibody (blood)	Meta-analysis	China [21]
Cholangiocarcinoma	Clonorchis sinensis	4.7 (2.2–9.8)	4.7 (2.2–9.8)	Fluke egg (stool)	Meta-analysis	Korea [22]
	HBV	2.7 (2.0–3.6)	2.7 (2.0–3.6)	HBsAg (blood)	Meta-analysis	International (mainly Asian) [23] 3387/98 428
	HCV	5.2 (2.1–12.8)	5.2 (2.1–12.8)	Anti-HCV antibody or HCV RNA (blood)	Case-control study	USA [24] 625/90 834
	*Opisthorchis* *viverrini*	14.1	14.1	Fluke egg (feces)	Cross-section study	Thailand [25] 12 311
Vulva	HPV	—	PAF 43	HPV DNA (tumor tissue)	Meta-analysis	International [20]
Vagina	HPV	—	PAF 70	HPV DNA (tumor tissue)	Meta-analysis	International [20]
Cervix uteri	HPV	—	PAF 100	HPV DNA (tumor tissue)	Meta-analysis	International [20]
Penis	HPV	PAF 50	—	HPV DNA (tumor tissue)	Meta-analysis	International [20]
Hodgkin’s lymphoma	EBV	PAF 46	PAF 46	EBV-DNA (tumor tissue)	Review	International [4]
Non-Hodgkin’s lymphoma	HIV-1	37.4 (36.0–39.0)	54.6 (16.1–60.7)	Not informed	Case-control study	USA [26] 2434/110 295
	HCV	2.5 (2.1–3.0)	2.5 (2.1–3.0)	Anti-HCV antibody and/or HCV RNA (blood)	Meta-analysis	International [27] 4169/6997
Burkitt’s lymphoma	EBV	3.9 (2.3–6.8)	3.9 (2.3–6.8)	VCA IgG (blood)	Case-control study	Uganda [28] 173/102
Kaposi’s sarcoma	KSHV/HIV-1	PAF 100	PAF 100	Not informed	Review	International [15]

EBV, Epstein-Barr virus; HBV, hepatitis B virus; HCV, hepatitis C virus; HIV-1, human immunodeficiency virus type 1; HPV, human papillomavirus; KSHV, Kaposi’s sarcoma herpes virus (also known as Human herpes virus type 8).

^a^PAF is assumed to be 100 in China (high nasopharyngeal carcinoma rate).

**Table 3.** Gender-specific prevalence of infectious agents in the general population and data source, by country

Country	HPV	*Helicobacter pylori*		HIV-1		HCV		HBV		Source
				
	Female %	Male %	Female %	Male %	Female %	Male %	Female %	Male %	Female %	
Korea	8.5	47.2	45.9	0.03^a^	0.03^a^	1.7	2.2	9.1	7.1	[29][30][12][31][12]
China	17.7	56.2^a^	56.2^a^	0.04	0.01	3.1	3.3	11.3	8.2	[32][33][34][35]
Japan	11.0	65.1^a^	65.1^a^	<0.1^a^	<0.1^a^	0.9^a^	0.9^a^	0.7	0.5	[36][37][38][39][40]
Indonesia	11.4	12.8^b^	12.8^b^	<0.1^a^	<0.1^a^	2.1^a^	2.1^a^	5.5^a^	5.5^a^	[41][42][43][44][38][45]
Philippines	4.4	71.9^b^	71.9^b^	<0.1^a^	<0.1^a^	0.4^a^	0.4^a^	13.3^a^	13.3^a^	[46][47][38][48][49]
Thailand	6.3	53.7^a^	53.7^a^	2.0^a^	2.0^a^	1.7^a^	1.7^a^	10.0	8.0	[50][51][38][52][53]
Vietnam	8.6	72.0	76.6	<0.1^a^	<0.1^a^	2.9^a^	2.9^a^	15.0	10.7	[54][55][38][56][57]
Bangladesh	NA	91.7^a^	91.7^a^	<0.1^a^	<0.1^a^	0.5^a^	0.5^a^	6.7	5.9	[58][38][59][60]
India	7.0	75.8^a^	75.8^a^	0.4^a^	0.4^a^	1.5^a^	1.5^a^	4.0^a^	4.0^a^	[61][62][38][63][64]
Iran	6.8	59.5	47.7	<0.1^a^	<0.1^a^	0.3^a^	0.3^a^	1.9	1.5	[65][66][67][68]
Pakistan	2.8	73.5	75.4	<0.1^a^	<0.1^a^	4.7^a^	4.7^a^	5.0^a^	5.0^a^	[69][70][38][71][72]
Turkey	4.2	51.8^a^	51.8^a^	<0.1^a^	<0.1^a^	1.2	1.8	6.5^a^	6.5^a^	[73][74][38][75][76]
Saudi-Arabia	5.6	75.8^a^	75.8^a^	<0.1^a^	<0.1^a^	5.1^a^	5.1^a^	2.6^a^	2.6^a^	[77][78][38][79][80]

HBV, hepatitis B virus; HCV, hepatitis C virus; HIV-1, human immunodeficiency virus type 1; HPV, human papillomavirus. NA, no available data.

^a^Sex-stratified prevalence was replaced by overall prevalence.

^b^The prevalence was among patients. Formula b was used when calculating PAFs.

**Table 4.** The PAFs of infectious agents and cancer sites in 13 Asian countries, by gender

Cancer sites	Infectious agents	Male	Female
Oral cavity	HPV	0%–15%	0%–15%
Oropharynx	HPV	1%–37%	1%–37%
Nasopharynx	EBV	90%–100%	90%–100%
Noncardia gastric cancer	*Helicobacter pylori*	11%–82%	11%–82%
Cardia gastric cancer	*Helicobacter pylori*	7%–33%	7%–35%
Anus	HPV	88%–90%	88%–90%
Hepatocellular carcinoma	HBV	11%–72%	8%–69%
	HCV	3%–38%	3%–38%
Cholangiocarcinoma	*Clonorchis sinensis*	1%–70%	1%–70%
	HBV	1%–18%	1%–18%
	HCV	1%–18%	1%–18%
Penis	HPV	50%	
Vulva	HPV		43%
Vagina	HPV		70%
Cervix uteri	HPV		100%
Hodgkin’s lymphoma	EBV	46%	46%
Non-Hodgkin’s lymphoma	HIV-1	1%–42%	0%–52%
	HCV	0%–7%	0%–7%
Burkitt’s lymphoma	EBV	20%	20%
Kaposi’s sarcoma	KSHV/HIV	100%	100%

EBV, Epstein-Barr virus; HBV, hepatitis B virus; HCV, hepatitis C virus; HIV-1, human immunodeficiency virus type 1; HPV, human papillomavirus; KSHV, Kaposi’s sarcoma herpes virus (also known as Human herpes virus type 8).

**Table 5.** Estimated new cancer cases and deaths caused by infection in 2012, by country

Country	Estimated new cases caused by infection			All new	Percentage %	Estimated deaths caused by infection			All cancer-related deaths^a^	Percentage %
		
	Male	Female	Total	cancer cases^a^		Male	Female	Total		
Korea	24 820	14 358	39 178	39 178	17.8%	10 810	5986	16 796	81 510	20.6%
China	444 493	233 869	678 207	678 362	22.1%	383 591	183 257	566 848	2 205 946	25.7%
Japan	57 409	37 710	95 119	95 119	13.5%	27 307	17 569	44 876	378 636	11.9%
Indonesia	18 626	28 778	47 404	47 404	15.8%	15 649	15 133	30 783	194 528	15.8%
Philippines	5581	9511	15 091	15 091	15.4%	4681	5115	9796	59 012	16.6%
Thailand	10 681	13 376	24 057	24 057	19.4%	9270	8713	17 984	84 981	21.2%
Vietnam	25 951	15 098	41 049	41 049	32.8%	23 152	11 036	34 188	94 743	36.1%
Bangladesh	5882	15 220	21 102	21 102	17.2%	5197	9471	14 668	91 339	16.1%
India	60 415	153 094	213 509	213 509	21.0%	52 251	93 076	145 327	682 830	21.3%
Iran	5402	3622	9024	9024	10.6%	4321	2460	6781	53 350	12.7%
Pakistan	5733	8711	14 444	14 444	9.8%	4917	5790	10 707	101 113	10.6%
Turkey	6109	5758	11 867	11 867	8.0%	4774	3885	8659	91 826	9.4%
Saudi Arabia	1021	799	1819	1819	10.4%	687	449	1136	9134	12.4%

NA, data not available.

^a^Excluding non-melanoma skin cancer (C44).

### Overview

Among six principle infectious agents, the most new cancer cases were attributed to HP (31.5%), followed by HBV (28.6%), and HPV (22.0%). This trend was slightly different for deaths; 32.8% of deaths from cancer attributed to major infectious agents were attributed to HP, while 27.3% of such deaths were attributed to HPV and 23.8% to HBV. Overall, the principle infectious agents caused 19.6% of new cancer cases and 22.0% of cancer deaths in the study areas. Figure illustrates different patterns of cancer incidence and death caused by infectious agents in both sexes.

**Figure.**
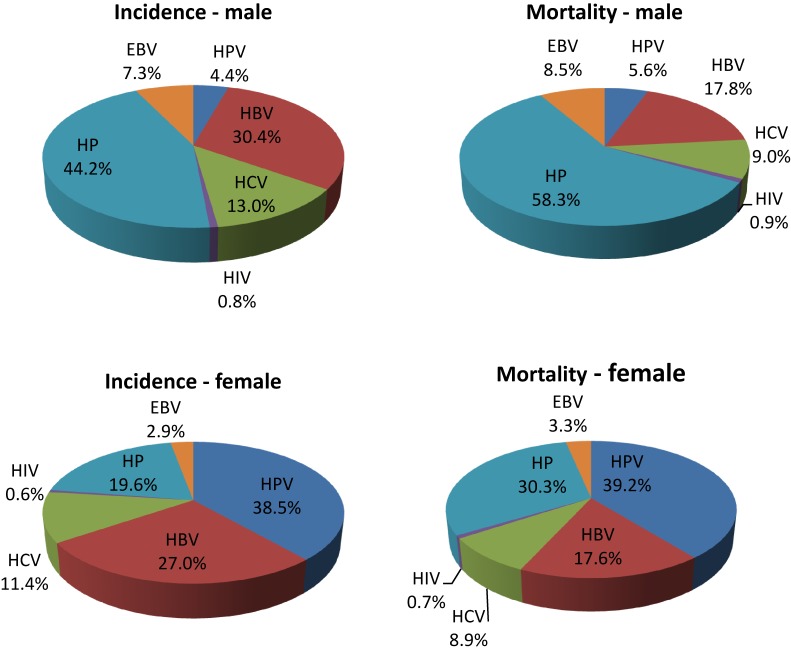
Proportion of estimated new cancer cases and deaths attributable to different infectious agents in men and women.

### Human papillomavirus

The high-risk genotypes (mainly types 16 and 18) of human papillomavirus are causative agents of up to 100% of cervical cancers, around 50% of penile cancers, 70% of vaginal cancers, 43% of vulvar cancers, 88% of anal cancers, 25% of oral cancers, and 35% of oropharyngeal cancers (especially lingual tonsillar cancers). Therefore, the PAFs of cervical cancer, anal cancer, penile cancer, vaginal cancer, and vulvar cancer were set at 100, 88, 50, 70, and 43, respectively.^14^ When it comes to oral cavity and oropharyngeal cancers, the correlations are less remarkable (HPV is positively detected in 20%–40% of cases^81^), and PAFs varied with prevalence of high-risk genotypes of HPV in different countries. HPV contributed to 29 324 and 275 429 new cancer cases and 20 613 and 141 612 deaths in men and women, respectively. Table 6 illustrates the new cancer cases and deaths due to HPV infection.

**Table 6.** Estimated new cancer cases and deaths attributed to HPV in 2012, by country

Country		Oral cavity		Oropharynx		Anus		Penis	Cervix uteri	Vulva	Vagina	Percentage^a^
						
		Male	Female	Male	Female	Male	Female	Male	Female	Female	Female	
Korea	New cases	29	18	99	10	153	157	42	3299	59	66	61.01%
	Deaths	10	5	44	4	39	46	18	1113	17	19	58.97%
China	New cases	2054	1167	2007	398	1193	876	1529	61 691	1171	1028	73.68%
	Deaths	1108	596	1312	333	644	494	1199	29 526	732	642	70.86%
Japan	New cases	484	339	1300	158	443	386	169	9390	246	186	51.81%
	Deaths	217	179	737	97	179	187	91	2712	131	99	42.53%
Indonesia	New cases	307	238	404	152	152	157	339	20 928	160	169	76.86%
	Deaths	129	101	321	116	100	104	251	9498	91	97	74.88%
Philippines	New cases	53	47	101	52	46	43	47	6670	45	56	70.12%
	Deaths	22	19	84	46	27	24	33	2832	23	29	62.17%
Thailand	New cases	128	92	187	37	59	77	212	8184	177	66	64.55%
	Deaths	66	47	118	23	35	46	162	4513	107	40	64.74%
Vietnam	New cases	110	60	251	46	43	56	172	5146	54	57	64.68%
	Deaths	50	27	202	36	30	38	144	2423	36	38	60.83%
Bangladesh	New cases	214	103	972	223	180	122	172	11 956	54	57	42.14%
	Deaths	123	59	831	191	132	90	144	6582	36	38	37.66%
India	New cases	3536	1521	5976	1310	2571	1863	2564	122 844	806	1962	57.21%
	Deaths	2393	1027	5085	1089	1937	1412	1915	67 477	489	1190	51.61%
Iran	New cases	49	39	23	17	147	85	0	947	7	35	46.94%
	Deaths	16	13	16	12	87	50	0	370	4	20	50.39%
Pakistan	New cases	20	16	17	8	219	149	341	5233	127	309	28.58%
	Deaths	11	9	14	7	159	110	260	2876	79	192	27.09%
Turkey	New cases	38	23	39	11	89	79	10	1686	93	42	51.68%
	Deaths	13	8	28	8	53	47	7	663	49	23	51.97%
Saudi Arabia	New cases	10	9	6	5	16	22	2	241	4	7	43.75%
	Deaths	3	2	4	3	9	12	1	84	2	3	47.31%

^a^The proportion of HPV-induced new cancer cases/deaths in all corresponding new cases/deaths.

### Epstein-Barr virus

The PAF of nasopharyngeal carcinoma caused by EBV was 90 in most Asian regions.^4^ In Southern China, an area with a high nasopharyngeal carcinoma burden, the fraction was assumed to be 100, which means that all cases of nasopharyngeal cancer and related deaths were attributable to EBV. According to the GLOBOCAN 2012 report,^82^ EBV resulted in 59 553 new nasopharyngeal cancer cases and 35 862 deaths in the target countries, with a gender ratio around 2:1. The top three countries that have the greatest burden of nasopharyngeal cancer caused by EBV in 2012 were China (33 198 new cases and 20 404 deaths), Indonesia (11 776 new cases and 6652 deaths), and Vietnam (4438 new cases and 2597 deaths). Furthermore, 8913 new cases and 6724 deaths related to Hodgkin’s lymphoma were attributed to EBV in 2012. As for EBV-related Burkitt’s lymphoma, there were 772 new cases and 464 deaths in the 13 countries analyzed.

### Human immunodeficiency virus type 1

The early 1990s saw the initial proliferation of human immunodeficiency virus type 1 (HIV-1) in Asia, and the seroprevalence of HIV in Asian countries was consequently low (<0.1%). Cases were sporadic, except for several countries and sites, such as Thailand and India, which experienced a relatively high burden of HIV (0.22%–2%).^38^^,^^83^^,^^84^ In 2012, 8089 NHL cases and 929 Kaposi’s sarcoma cases were attributed to HIV infection, with 5227 and 494 respective deaths. In India, there were 3417 new NHL cases and 19 new Kaposi’s sarcoma cases, making India the country with the highest HIV-related cancer burden in Asia. Although the HIV prevalence rate in the general population was highest in Thailand, there was less overall burden (39 new Kaposi’s sarcoma cases and 1832 new NHL cases) because of its small total population. Although most cases of anal cancer were related to high-risk type HPV, HIV also contributed to the anal cancer burden, due to immune suppression caused by the HIV infection.

### Hepatitis B virus and hepatitis C virus

Asia has a heavy disease burden from hepatitis B virus (HBV) and hepatitis C virus (HCV), both of which contribute to hepatocellular carcinoma and cholangiocarcinoma. Moreover, HCV is related to NHL. Table 7 lists the numbers of new cases and deaths from cancers resulting from HCV and HBV infection in the target countries.

**Table 7.** Estimated new cancer cases and death caused by HBV and HCV in 2012, by country

Country		Hepatocellular carcinoma HBV-related		Cholangio- carcinoma HBV-related		Hepatocellular carcinoma HCV-related		Cholangio- carcinoma HCV-related		Non-Hodgkin’s lymphoma HCV-related		Percentage^a^
				
		Male	Female	Male	Female	Male	Female	Male	Female	Male	Female	
Korea	New cases	6670	2428	355	90	1481	654	38	16	64	68	54.88%
	Deaths	3824	1330	461	120	849	358	50	21	23	22	50.83%
China	New cases	154 616	47 390	6965	1829	64 048	23 174	5069	1852	1160	778	70.17%
	Deaths	148 545	47 366	6692	1828	61 533	23 162	4870	1852	738	466	72.51%
Japan	New cases	2461	975	12	5	2163	1145	37	20	144	132	12.41%
	Deaths	2178	828	11	4	1914	972	33	17	80	67	14.29%
Indonesia	New cases	5174	1841	168	60	2168	771	162	58	237	170	34.37%
	Deaths	4899	1750	159	57	2052	733	154	55	31	115	45.61%
Philippines	New cases	3021	1273	148	62	211	89	14	6	8	7	48.14%
	Deaths	2916	1211	142	59	203	84	14	6	5	4	52.43%
Thailand	New cases	3902	1385	1217	389	1077	418	582	226	56	44	38.09%
	Deaths	3705	1320	1156	370	1023	398	553	215	39	30	39.77%
Vietnam	New cases	9671	2678	503	117	3496	1077	274	84	65	46	73.01%
	Deaths	9199	2545	478	111	3326	1024	260	80	45	32	75.11%
Bangladesh	New cases	781	475	27	16	84	54	6	4	11	6	27.58%
	Deaths	741	455	26	15	80	52	5	3	8	5	30.33%
India	New cases	5591	3302	161	95	2108	1245	152	90	347	173	25.90%
	Deaths	5435	3246	156	93	2049	1224	148	88	242	121	29.52%
Iran	New cases	174	110	4	2	21	16	1	1	7	5	7.07%
	Deaths	166	105	4	2	20	15	1	1	5	3	9.26%
Pakistan	New cases	1040	552	32	17	819	435	70	37	245	148	33.00%
	Deaths	987	528	31	16	778	416	66	35	179	109	37.05%
Turkey	New cases	646	290	22	10	156	99	11	7	51	56	18.63%
	Deaths	613	277	21	10	148	94	10	7	31	34	23.94%
Saudi Arabia	New cases	126	50	3	1	157	63	14	5	51	37	25.76%
	Deaths	118	48	3	1	148	60	13	5	28	21	32.39%

HBV, hepatitis B virus; HCV, hepatitis C virus.

^a^The proportion of HBV- and HCV-induced new cancer cases/deaths in all these cancers cases/deaths.

### Helicobacter pylori

The cases and deaths of gastric cancer caused by HP are shown in Table 8.

**Table 8.** Estimated new cases and deaths of HP-related gastric cancer in 2012, by country

Country	Noncardia gastric cancer				Cardia gastric cancer				Percentage^a^	
		
	New cases		Deaths		New cases		Deaths		New cases	Deaths
			
	Male	Female	Male	Female	Male	Female	Male	Female		
Korea	13 705	6880	4439	2657	1404	367	455	142	71.50%	71.59%
China	166 389	78 450	129 994	66 944	14 122	3632	11 033	3099	64.84%	64.91%
Japan	45 048	22 728	19 613	11 337	4104	1129	1787	563	67.66%	67.78%
Indonesia	324	203	292	183	54	20	48	18	10.00%	10.01%
Philippines	673	523	571	441	75	35	64	29	54.08%	54.09%
Thailand	933	776	751	625	77	39	249	31	64.24%	72.44%
Vietnam	5863	3295	5355	2981	561	194	512	176	69.80%	69.79%
Bangladesh	2653	1799	2497	1675	285	115	268	107	73.70%	73.70%
India	27 346	13 511	25 666	12 557	2681	792	2517	736	70.26%	70.25%
Iran	3955	1840	3374	1573	345	86	294	74	64.45%	64.45%
Pakistan	1508	980	1408	913	146	57	136	53	70.08%	70.05%
Turkey	3408	2610	2918	2232	278	127	238	109	63.47%	63.46%
Saudi Arabia	212	134	172	110	21	8	17	6	70.62%	70.28%

^a^The proportion of HP-related gastric cancer cases/deaths in all gastric cancer cases/deaths.

## DISCUSSION

The present study is the first to assess the burden of potentially oncogenic infections and their related cancers in Asia. Based on the methods of previous studies in China^85^ and Korea,^11^ the nation-specific data of prevalence of infections by oncogenic agents and the general population RRs of infection-related cancers were collected for PAF calculation. Additionally, we estimated the cancer burden attributable to infection in 13 Asian countries using the numbers of new cancer cases and deaths provided in GLOBOCAN2012. Overall, we estimated that 1 212 026 new cancer cases (19.6% of all cancers) and 908 549 deaths (22.0% of all cancer-related deaths) were caused by infection in 2012. Parkin’s study in 2002^4^ estimated that infectious agents were responsible for 17.8% of cancer cases worldwide (26.3% of cases in developing countries and 7.7% of cases in developed countries). Our results were somewhat different from previous nation-specific PAF calculations^11^^,^^85^ because most of the RRs in our study were summarized from international studies that were generalizable to different Asian countries; for example, we chose the RR of 18.8 for HBV, which was taken from a study with a large sample size,^21^ rather than the RRs in Shin’s study^11^ (24.45 in men and 33.73 in women), which were taken from a meta-analysis of Korean data and only represented the Korean population. Moreover, we estimated the number of cancers based on GLOBOCAN2012, while previous studies used Korean data from 2007. This earlier data found lower prevalence than those of GLOBOCAN2012, and the proportion of infection-related cancers in all cancers was consequently lower. However, our results are consistent with Parkin’s estimates for developing countries because the majority of countries in our study are developing countries (except for Japan and Korea). Specifically, 20.5% of new cancer cases were related to infection in the 11 developing Asian countries, and 14.5% of new cancer cases were related to infection in Korea and Japan. Because the background prevalence of infectious agents in Korea and Japan differed from other developed areas (mainly Europe and North America), the proportion of infection-related cancers in developed countries was doubled in our study.

Another study in 2008^3^ revealed that 16.1% of new cancer cases were attributed to infection worldwide, and the proportion was higher in less developed countries (22.9%) than in more developed countries (7.4%). While both Parkin’s and Martel’s studies focused on global infection-related cancer burden, our study is designed in a region-specific way, in which we analyzed the data from 13 Asian countries to illustrate the infection background of cancer cases and deaths in Asia in 2012. The findings of our study provide comparable data for different Asian countries, which enable us to gain insight into the regional status of infectious agents that are linked to cancer incidence. The potential burden of disease (BOD) described in the Asian region could be significantly ameliorated with intervention measures, such as prophylactic vaccinations for HBV and HPV. In addition, this description of the BOD offers a baseline reference for governments seeking to address the public health issues of infectious disease and cancer. Combining the outcomes of the current study with the data of medical expenses and living expenditures of corresponding countries, the economic burden caused by infection could be calculated. These economic burden calculations can illustrate to policymakers the importance of taking immediate action on infection control for these agents, which may improve the living standard of the public, reduce unnecessary loss of lives, and reduce financial burden.

The gender-specific patterns of incidence and mortality caused by different infectious agents (shown in Figure) are of practical consequence when designing preventive interventions and treatment for targeted population. For men, HP and HBV are the main causative factors of gastric cancer and liver cancer, respectively, together comprising over three quarters of infection-related cancer cases and deaths. HBV infection contributes to twice the liver cancer burden in men as in women, and much work remains to reduce the high prevalence of HBV in Southeast/East Asia. In women, HPV and HP were associated with heavy burden of cervical and gastric cancers, respectively. These findings indicate that more attention should be paid to prevention of HPV in women, and both prevention and treatment of HP play vital roles in reducing cancer burden in Asia for both genders.

### HIV

Although infection with HIV alone will not cause many common HIV-associated cancers, it has been recognized as a major contributing factor to some specific cancers because cancer incidence rises significantly with combined infection of HIV and strong cancer-causing infectious agents. The carcinogenic mechanisms of HIV are immune deficiency and suppression, which lead to the failure of immunological surveillance of tumor cells and susceptibility to carcinogenic factors. The contribution of HIV to the development of many cancers is complicated; for example, HIV can increase the risk of anal cancer, which is caused by HPV. To avoid over-counting for these co-infections, we only estimated cases and deaths of Kaposi’s carcinoma and NHL, in which HIV plays a main role, though in comparison to European or North American countries, the prevalence of Kaposi’s carcinoma in Asia is quite low.^15^ The numbers of cancer cases and deaths related to HIV infection might be overestimated because we considered the estimated prevalence of HIV in low-prevalence areas as 0.1%, while UNAIDS^38^ reported prevalence below 0.1% in the general population. The current study estimates that HIV contributed to 9018 new cases and 5721 deaths from cancer in the study area. However, the rates of HIV infection started to rise after the low-prevalence stage of the early 1990s, increasing from 0.1% to 1.3% in 2009.^83^ The pattern of HIV spread in Asia has gradually changed, from sporadic new cases in the general population and epidemic in high-risk groups to a low-level epidemic in the general population. As a consequence, the numbers of HIV-associate cancer cases and deaths is expected to increase in a few years, especially in regions such as Thailand and India. The HIV prevalence of these areas is much higher than in the rest of the surveyed Asian countries, which is expected to cause health and social problems, including short lifespan and poverty. Fortunately, the utilization of antiretroviral therapy and behavioral interventions in Asian areas have had initial success in reducing costs of HIV control.^83^ However, much work is still required to control the spread of HIV in developing countries.

### HP

There has been controversy over the direct evidence linking HP infection and gastric cancer in Asia,^78^ where there is some doubt that the high infection rate of HP (eg, in Japan and Bangladesh^58^) is a predictor of the high prevalence of gastric cancer. At an individual level, HP has been associated with the occurrence of gastric cancer, but some Asian countries have a low prevalence of gastric cancer despite a high HP prevalence. Alcohol and cigarette consumption in these areas may cause many of these gastric cancers. However, a Japanese study^40^ revealed that both groups testing strongly positive and weakly positive for HP antibody showed significantly higher incidence of gastric cancer compared to those testing negative. We adopted a combined analysis, which illustrated that the OR for non-cardia gastric cancer and HP was 5.9 (95% CI, 3.4–10.3) compared with those testing negative for HP,^18^ while the relationship was less significant between cardia gastric cancer and HP infection (RR1.6; 95% CI, 1.0–2.5).^19^ Consequently, we estimated that approximately 436 501 new cancer cases and 324 042 deaths were caused by HP in 2012. The use of regular and rational combinations of antibiotics is the optimal secondary prevention strategy against HP infection, but such use of antibiotics is the primary prevention method for gastric cancer. Using regular gastroscope screening tests, early lesions on the gastric epithelium can be detected before they progress to cancer.^86^

### HPV

In our study, new cancers in the oral cavity, oropharynx, cervix, vulva, and vagina that were associated with high-risk types of HPV totaled 278 403 cases, ranking HPV the highest among the principle infectious cancer-causing agents in women. HPV was also estimated to be responsible for 162 762 cancer deaths in Asia, making HPV the second-ranked infectious factor leading to cancer deaths. To estimate the burden of HPV-related vulvar, vaginal, penile, and anal cancers, we applied a geographic similarity method in cases where country-level data was not available in CI5X. Namely, neighboring countries’ data were used to substitute for the corresponding incidence or mortality of the country with missing data. Admittedly, this method has some limitations, because the incidence and mortality of cancers vary by country. However, it is likely to be a more accurate way of estimating the sub-category cancer burden with the present database than by assuming the same incidence and mortality across Asia without considering geographic factors.

Since the currently licensed prophylactic HPV vaccines (Gardasil for type 6, 11, 16, and 18 and Cervarix for type 16 and 18) marketed in North America, Europe, Australia,^87^ and some parts of Asia became available, the expectation is that HPV-related cancers maybe largely prevented if these vaccines are adopted with high coverage. Both vaccines have been shown to be effective, immunogenic, and safe.^88^ However, the vaccines have not been approved to market in most areas of Asia. Where they are approved, such as Hong Kong and Korea, they are high-priced and less likely to be listed in social medical insurance, therefore largely only available to those who can afford them. Where governments have endorsed and paid for vaccines, such as in Australia,^87^ reductions in rates of HPV-related infections^89^ and high-grade lesions (ie, lesions coded as cervical intraepithelial neoplasia of grade 2 or worse or adenocarcinoma in situ)^90^ have already been seen. The vaccines are currently accessible through school-based government-funded programs only in a few regions, such as Malaysia, Bhutan, and Japan. The predicted outcome is that the HPV-related cancer burden will decline if the primary prevention measure of HPV vaccines is applied in the general population by governments. In addition, screening with cytology tests, visual inspection, or HPV-DNA detection are practical secondary prevention methods that can be employed against cervical cancer in low-resource countries and areas.

### HCV and HBV

HCV and HBV were highly epidemic up to the early 1990s in many areas of Asia, resulting in a relatively high incidence of liver cancer decades later. However, the late 1990s saw the introduction of the HBV vaccine among all newborns, particularly babies of women who were chronically infected with HBV. Widespread HBV vaccination has been markedly effective in reducing the HBV epidemic in areas where good coverage has occurred. A Taiwanese study showed that universal HBV vaccination for newborns provided long-term protection for up to 20 years, which enabled prevention of the infection before adulthood^91^; meanwhile, obligatory screening of blood and organ donations has significantly curbed HCV transmission. Together, HBV and HCV caused 431 086 new liver cancer cases and 406 779 deaths in 2012, representing 77.61% of liver cancer cases and 76.6% of deaths. In addition, liver flukes, such as *Clonorchis sinensis* and *Opisthorchis viverrini*, were also causal factors of liver cancer (responsible for 1701 new cholangiocarcinoma cases in our estimation). *C. sinensis* is still an endemic parasite in areas of river basins in East Asia, while *O. viverrini* remains epidemic in Thailand.^4^^,^^92^ In this case, improving public awareness of the risk of the consumption of raw or insufficiently cooked fish, management of fecal sewage, and the supervision of intermediate hosts in epidemic areas are of great importance in controlling fluke infection and preventing related liver cancers.

Inevitably, our study has some limitations. First, not all prevalence data of infectious agents in the target countries were available. For instance, the prevalence of EBV in most countries and the prevalence of HPV in Bangladesh were not readily available from the databases to which we had access. We used overall PAFs for the cancers caused by EBV in all target countries and PAFs of HPV in Bangladesh. Moreover, the RRs we adopted are not country-specific, except for some RRs taken from independent studies on infection-related cancer burden in Korea^10^ and China.^85^ Numbers of cancer deaths in Japan were taken from the data of Monitoring Cancer Incidence in Japan (MCIJ) Project. The mortality data reported in MCIJ were lower than those in GLOBOCAN 2012, and we recognized them as more reliable and valid due to the wider regions that the program covered and the high-quality systematic registration method the MCIJ Project employed. According to the proportion of cancer deaths, some cancer categories in the MCIJ report were split into the corresponding sub-categories, as in GLOBOCAN, to facilitate estimation. As we were focusing on estimated numbers of infection-related cancer cases and deaths rather than comparing cancer incidence and mortality among different countries, we did not do age or sex standardization when using two data sources. Age-standardized data were available in both the MCIJ and GLOBOCAN. Old research was used to extrapolate the prevalence within a defined period, which affected the results because older technology for detection of infectious agents was limited. In addition, the geographic similarity assumption was utilized to tackle the inaccessible proportions of other and unspecified cancers (eg, penile, vulvar, and anal cancer) of the countries not included in CI5X. The data of CI5X came from a different time period (1993–1997), and the rest of data were from 1997 to 1999. Some of the RRs were extracted from non-Asian studies if the data could not be obtained in reliable Asian studies. As a consequence, the estimations in our study ignored some geographic variation among these countries. There might be some systematic differences between larger and smaller countries in Asia. In addition, the sample sizes of some prevalence studies of infectious agents in some countries were small or contained severe bias because the subjects were blood donors or hospital-based populations rather than general populations, which makes it hard to extract valid prevalence. For example, the majority of studies of HBV and HCV prevalence in Japan were based on the registry system of blood donation,^39^^,^^40^ resulting in limited representativeness. As liver flukes are endemic in a limited area in Asia and accurate data were not available, rates of cholangiocarcinoma and urinary bladder cancer caused by liver flukes were not included in our study. The exclusions of cholangiocarcinoma and urinary bladder cancers in calculating the prevalence of cancers and deaths may have caused a slight underestimation of the deaths and new cases attributable to this infection.

In conclusion, infectious factors play a major role in the etiology and progression of various cancers, which contributes to about one quarter of all cancer cases and cancer-related deaths. Infection by these agents not only results in the loss of life, but also imposes heavy economic burden on families and societies, both directly and indirectly. Adopting long-term measures (including primary and secondary prevention) to prevent infection from the principal cancer-causing agents is an efficient way to reduce rates of infection-related cancers. Tools are available to prevent many of these diseases and should be utilized.
